# Development of Nanopore amplicon sequencing method for culture-free genotyping of *Bacillus anthracis* strains directly from environmental samples

**DOI:** 10.3389/fmicb.2026.1771578

**Published:** 2026-03-13

**Authors:** Ágnes Nagy, Gábor Endre Tóth, Péter Sály, Csaba István Pereszlényi, Gergely Csaba Babinszky, László Makrai, Balázs Antal Somogyi, Miklós Gyuranecz

**Affiliations:** 1 Hungarian Defence Forces Medical Centre, Budapest, Hungary; 2 Virus Metagenomics and Evolution Group, Bernhard Nocht Institute for Tropical Medicine, Hamburg, Germany; 3 Department of Arbovirology and Entomology, Bernhard Nocht Institute for Tropical Medicine, Hamburg, Germany; 4 HUN-REN Centre for Ecological Research, Institute of Aquatic Ecology, Budapest, Hungary; 5 Department of Fisheries Research and Development, Institute of Aquaculture and Environmental Safety, Hungarian University of Agriculture and Life Sciences, Gödöllő, Hungary; 6 Autovakcina Kft., Budapest, Hungary; 7 National Laboratory of Virology, University of Pécs, Pécs, Hungary; 8 HUN-REN Veterinary Medical Research Institute, Budapest, Hungary; 9 National Laboratory of Health Safety, Budapest, Hungary; 10 MolliScience Kft., Biatorbágy, Hungary; 11 University of Veterinary Medicine, Budapest, Hungary

**Keywords:** *Bacillus anthracis*, Central Europe, culture-free genotyping, environmental samples, genetic diversity, Hungary, MLVA31, Nanopore amplicon sequencing

## Abstract

Fast and accurate genetic subtyping of pathogens is required to respond appropriately to biological events caused by natural outbreaks or bioattacks involving anthrax. In this study, we developed and validated a culture-free genotyping method that combines a multiplex PCR-based amplicon sequencing method on the Nanopore platform with *in silico* multiple-locus variable-number tandem repeat analysis (MLVA) of 31 loci to identify an unknown *Bacillus anthracis* strain directly from environmental samples. The novel method accurately identified repeat numbers for all loci in 12 different MLVA genotype *Bacillus anthracis* strains analyzed in the study, matching 100% with the reference capillary electrophoresis and Sanger sequencing results. The detection limit of the method, at which all 31 variable-number tandem repeat loci were successfully identified, was found to be 10^4^ CFU spores/sample for pure spore samples and at 10^6^ CFU spores/sample for spiked environmental samples from three matrices (soil, swab, and muddy water). Specificity tests yielded negative results for samples containing only non-*Bacillus anthracis* members of the *Bacillus cereus* group, which produced sequencing reads for 15 loci but were non-specific to *Bacillus anthracis.* To validate the method, we genotyped 11 *Bacillus anthracis* strains originating from a historical collection of Hungarian isolates. The MLVA31 typing scheme classified the strains into five groups, four of which fell into the A.Br.008/009 Trans-Eurasian (TEA) group within the clade A, and one into the B.Br.CNEVA group within the clade B. The largest group within clade A comprises six strains that are assumed to be members of the dominant *Bacillus anthracis* population in Hungary. Our results demonstrate that PCR-based amplicon sequencing using the portable MinION device is highly effective for on-site genotyping of pathogens directly from environmental samples. This establishes the NGS-based MLVA genotyping as a valuable tool for biodefense laboratories in preliminary forensic investigations of bioterrorism-related anthrax outbreaks. Furthermore, our results provide new insights into the genetic diversity of *Bacillus anthracis* in a region (Hungary, Central Europe) that is underrepresented in research and has limited scientific data.

## 1 Introduction

Genetic analysis and strain-level identification of *B. anthracis* isolates for accurate phylogenetic typing and continuous monitoring of the global distribution of naturally occurring strains are crucial from both an epidemiological perspective and in forensic investigations of suspected bioterrorism acts resulting in the deliberate release of the pathogen. *B. anthracis* belongs to the *Bacillus cereus* group, forming a distinct monomorphic cluster within it (Kolstø et al., 2009) having greater than 99% sequence identity between strains (Turingan et al., 2013). The extremely low genetic diversity of *B. anthracis* strains is attributed to the slow accumulation of mutations due to its highly specialized life cycle and short evolutionary history. The mutations emerge during DNA replication in the reproductive phases of rare and short outbreaks, while in the extended periods (years or even decades) between outbreaks, *B. anthracis* remains in a dormant, non-reproductive spore form in the soil (Keim et al., 2009). In environmental samples, the pathogen is only present in low quantities within the overall isolated DNA sample, which contains a high amount of non-target genetic material from various environmental microorganisms. Among these, particularly the presence of the *Bacillus cereus* group members complicates the precise strain-level genotyping of *B. anthracis* due to their genetic similarity (Irenge and Gala, 2012).

For genetic characterization of *B. anthracis* isolates the typing strategy called PHRANA (Progressive hierarchical resolving assays using nucleic acids) was developed (Keim et al., 2004). This is a nested hierarchical approach using three types of molecular markers, each with differing mutation rates and discriminatory power. The typing system is a combination of identifying 13 canonical single nucleotide polymorphisms (canSNP), analysis of 31 variable-number tandem repeat (VNTR) markers (multiple-locus variable-number tandem repeat analysis – MLVA), and finally genotyping 4 single nucleotide repeats (SNR). To determine canSNP, MLVA, and SNR genotypes in the pre-NGS (next-generation sequencing) era, PCR-based assays were applied. As NGS has become widely available and affordable, classical PCR-based typing approaches are being replaced by *in silico* analysis of data generated by whole-genome sequencing (WGS) (Brangsch et al., 2022). Genome-wide single-nucleotide polymorphisms (wgSNP) and core genome multilocus sequence typing (cgMLST) enable high-resolution clustering and detailed phylogenetic analysis of whole-genome sequences from unknown strains (Bruce et al., 2020; Abdel-Glil et al., 2021).

Accurate genetic characterization of *B. anthracis* using both PCR- and WGS-based methods prior to genotyping requires purification of isolates by culturing and identification of suspected colonies using *B. anthracis*-specific PCR assays (Kim et al., 2013; Braun et al., 2015; Rume et al., 2016; Timofeev et al., 2019; Pandiarajan et al., 2026), which is a labor-intensive and time-consuming workflow. Culture-free typing approaches, which are performed directly on environmental samples, are necessary to achieve the required speed of analysis. However, NGS can be performed directly on extracts from clinical (e.g., bodily fluids and tissues), food, or environmental (e.g., soil, surface swab, powder, sludge, and natural water) samples, the quality of DNA and the quantity of the pathogen DNA are often insufficient for generating high-quality whole genomes suitable for complete wgSNP or cgMLST genotyping (Dennis et al., 2022). Several applications exist for culture-free genotyping of pathogens directly from clinical and environmental samples using NGS, such as shotgun metagenomics, and various targeted sequencing methods. Shotgun metagenomics aims to sequence the complete genetic material present in a sample, thereby enabling the detection of all potential pathogens (Batool and Galloway-Peña, 2023), facilitating the characterization of pathogens with determination of antibiotic resistance profiles, virulence gene information, cgMLST and SNP profiles (Zhao et al., 2024). However, metagenomic sequencing is limited by a relatively low level of sensitivity, failing to detect low-abundance target genes from samples with low target content (Wang et al., 2025).

Targeted sequencing approaches, such as PCR amplicon sequencing, hybrid-capture target enrichment, CRISPR/Cas9 enrichment, and Oxford Nanopore adaptive sampling, offer sensitive and accurate identification of selected targets by enriching them prior to sequencing, making them more effective than shotgun metagenomics for detecting and identifying pathogens in samples that contain low target levels and high concentrations of background microorganisms (Zhao et al., 2024; Luo et al., 2025). A cost-effective, high-throughput, and high-sensitivity target sequencing technique is a method for sequencing multiple targets that have been amplified by multiplex PCR. This technique is widely used for the rapid and routine detection, identification, and molecular typing of bacteria. Although PCR amplicon sequencing offers high sensitivity through the effective enrichment of target genetic regions, it has limitations in terms of the number of targets and length of the regions covered. Primer dimer generation, heterogeneous amplification efficiency, and the effects of competitive amplification of multiple targets can all negatively impact sensitivity (Luo et al., 2025).

Amplification-free target enrichment techniques (hybrid-capture target enrichment, CRISPR/Cas9 enrichment and Oxford Nanopore adaptive sampling) are developed to overcome the limitations of amplicon-sequencing, enable non-PCR-based parallel enrichment of large numbers of targets (hundreds to thousands) and longer regions of target genes. These methods have been shown to provide accurate and high-resolution strain-level genotyping of pathogenic bacteria from complex sample types (Cottingham et al., 2025). Notwithstanding the clear advantages of these techniques in terms of genotyping resolution, there are several limitations to consider. The design of hybrid-capture target enrichment and CRISPR/Cas assays is complicated, assays are complex and time-consuming, requiring a multi-step enrichment and library preparation workflow, and these techniques are at high-cost (Cottingham et al., 2025; Luo et al., 2025; Quek and Ng, 2024).

In the context of biowarfare agents, main aim of culture-free applications of NGS, is to rapidly detect and unambiguously identify the pathogens at species level directly from environmental samples (Israeli et al., 2019; Player et al., 2020; Dias et al., 2026). For genotyping of *Bacillus anthracis* strains directly from environmental samples, shotghun metagenomics and hybrid-capture target enrichment were used (Podowski et al., 2025; Dennis et al., 2022). In earlier studies, metagenomic next-generation sequencing (NGS) was considered to be unsuitable for the precise strain-level genetic characterization of an unknown *B. anthracis* strain directly from environmental samples, due to the presence of closely related *B. cereus* species (Be et al., 2013). A recent study demonstrated that shotgun sequencing of complex samples is a viable method for identifying *B. anthracis* sublineage in outbreak tracking (Podowski et al., 2025). However, the genotyping accuracy was only satisfactory for samples with a high bacterial load (Podowski et al., 2025). Similarly, hybrid-capture target enrichment was demonstrated a sufficient depth of coverage for haploid variant calling in samples with higher pathogen-specific DNA concentration (Dennis et al., 2022).

The most suitable approach for genotyping *B. anthracis* isolates using the amplicon sequencing method is the analysis of 31 VNTR markers. MLVA has sufficient resolution to distinguish between two *B. anthracis* strains because of the high mutation rate (10^−5^ to 10^−4^ per generation) and a large number (tens) of possible allelic states of the VNTR regions (Keim et al., 2004; Birdsell et al., 2012; Thierry et al., 2014). Furthermore, MLVA enables the reliable identification of *B. anthracis* in samples containing other *B. cereus* group bacteria (Keim et al., 2000; Marston et al., 2006). Multiple MLVA schemes have been developed, using an increasing number of loci including MLVA8 (Keim et al., 2000), MLVA15 (Van Ert et al., 2007), MLVA25 (Lista et al., 2006), and MLVA31 (Beyer et al., 2012). The resolution of widely used MLVA schemes for *B. anthracis* genotyping is getting more sophisticated with the using of increasing number of loci examining the *B. anthracis* strains from the same region. The MLVA method for genotyping *B. anthracis* strains has been used routinely since 2000 (Keim et al., 2000) for genotyping and classification of *B. anthracis* strains isolated from one particular outbreak (Liu et al., 2017; Li et al., 2020; Metrailer et al., 2023) or from outbreaks on a national or provincial/county scale across different temporal periods, from decades to years (Antwerpen et al., 2011; Beyer et al., 2012; Durmaz et al., 2012; Thierry et al., 2014; Mao et al., 2016; Muller et al., 2020; Rondinone et al., 2020; Shevtsov et al., 2021; Lekota et al., 2024; Izbanova et al., 2025). The efficacy of the MLVA approach in identifying intentionally released *B. anthracis* strains was demonstrated in the context of microbial forensic investigations of the Aum Shinrikyo anthrax release in 1993 (Keim et al., 2001) and the Amerithrax attack in 2001 (Hoffmaster et al., 2002).

A substantial online database, designated MLVAbank (MLVABank for Microbes Genotyping, 2023), has been constructed using MLVA typing schemes, containing information regarding the MLVA profiles of over 3,000 *B. anthracis* strains isolated globally (Le Flèche et al., 2001; Grissa et al., 2008). The availability of third-generation NGS techniques has enabled the use of whole-genome sequence-based *in silico* MLVA for genotyping *B. anthracis* (Brangsch et al., 2022; Metrailer et al., 2023). This approach makes data comparable with that obtained using the reference capillary electrophoresis method for MLVA genotyping and deposited in the MLVABank database. Ensuring backward compatibility of WGS data for new isolates with MLVA data deposited in MLVABank or published elsewhere provides comparability with strain collections that were not whole genome sequenced (Linde et al., 2023).

For sequencing VNTR amplicons containing long repetitive elements, third-generation sequencers from Oxford Nanopore Technologies (ONT) and Pacific Biosciences (PacBio) are the most suitable among the currently available NGS platforms. Long reads generated by these sequencing techniques have the capacity to span the entire genome region containing repeat sequences, thus facilitating the assembly and size determination of these regions (Loman et al., 2015). PacBio’s error rate of less than 1% still outperforms that of Nanopore, which achieves a rate of 2–5% with the latest chemistry, flow cells and base-calling algorithms. However, compared to PacBio, ONT instruments offer advantages in terms of cost, size, portability, and field applicability (Chaushevska et al., 2025).

In this study we developed and validated a multiplex PCR-based amplicon sequencing assay with ONT MinION device and *in silico* VNTR analysis method for MLVA typing of *B. anthracis* directly from environmental samples. Our primary objective was to design and optimize a rapid and sensitive genotyping assay applicable in field laboratories to provide preliminary on-site information about the possible source of an unknown *Bacillus anthracis* strain in an outbreak caused by natural or deliberate release of the pathogen.

We optimized and evaluated our method on 12 *Bacillus anthracis* strains with different MLVA genotypes, 11 of which were retrieved from a historical strain collection isolated between 1930 and 2014 in Hungary. Anthrax is a rare disease in Hungary, occurring only sporadically. Between 2006 and 2023, 16 animal (Kozytska et al., 2023) and 15 human cutaneous anthrax cases (Nemzeti Népegészségügyi és Gyógyszerészeti Központ, 2023) were reported. Limited publicly available information exists regarding the genotyping and whole-genome sequencing data of Hungarian isolates. PCR-characterized canSNP genotypes of 29 historical strains (Kreizinger et al., 2016) and MLVA genotypes of three strains have been published (Keim et al., 2000; Van Ert et al., 2007). Through the development and testing of a novel *in silico* MLVA assay, this study facilitates the classification and examination of the possible origin of *B. anthracis* strains from an under-represented geographical region.

## 2 Materials and methods

**Figure 1:**
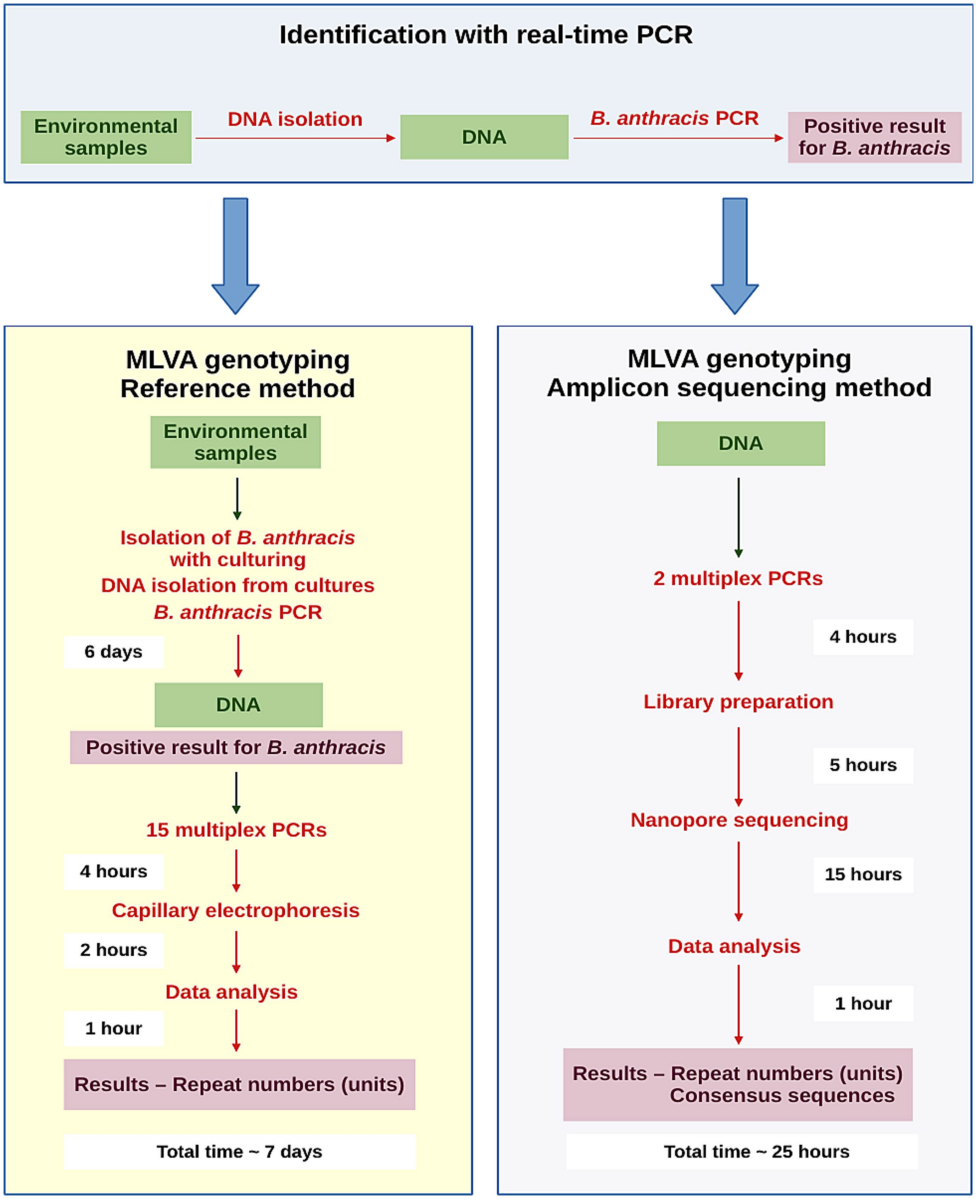
Workflow for the identification and MLVA genotyping of an unknown *Bacillus anthracis* strain from environmental samples using the reference capillary electrophoresis method and the novel amplicon-based NGS method.

An overview of the workflow of our novel NGS-based method, alongside capillary electrophoresis as the reference method used for *B. anthracis* MLVA analysis, is shown in Figure 1.

### 2.1 Strains, culturing, and spore production

All procedures involving the use of virulent *Bacillus anthracis* spores were performed in a biosafety level 3 (BSL3) laboratory of the Hungarian Defence Forces Medical Centre, Epidemiological and Scientific Research Institute.

Twelve *Bacillus anthracis* strains, one *Bacillus cereus,* and one *Bacillus thuringiensis* strain were used for the development and performance evaluation of the novel NGS-based MLVA typing method. Eleven wild-type virulent *B. anthracis* strains (Hungarian isolates BaML_1, BaML_4, BaML_7, BaML_9, BaML_10, BaML_16, BaML_36, BaML_39, BaML_49, BaML_52, and BaML_61 from animal outbreaks) and one non-virulent Sterne 34F2 vaccine strain were provided by the University of Veterinary Medicine Department of Microbiology and Infectious Diseases and the Hungarian Research Network Veterinary Medical Research Institute. The 11 Hungarian wild-type strains originated from a historical strain collection. However, apart from the basic information that all strains were isolated from infected animals during natural outbreaks in Hungary between 1930 and 2014, no records are available on the exact location or dates of isolation. The *B. cereus* strain ATCC 11778 was obtained from the Hungarian National Collection of Medical Bacteria, and the *B. thuringiensis* strain ATCC 33679 was obtained from the National Collection of Agricultural and Industrial Microorganisms.

DNAs from five different *B. cereus* group isolates were used for specificity testing of the assay: two *B. thuringiensis* (strain B509 and B511), one *B. mycoides* (strain B546), and two *B. weihenstephanensis* (strain B566 and B567) DNA templates were provided by the Defence Laboratories Department, Belgian Armed Forces.

The *B. anthracis, B. cereus* and *B. thuringiensis* strains were grown on Columbia blood agar plates (Merck, Darmstadt, Germany) at 37 °C. To obtain vegetative cell suspension, bacterial colonies were taken from 18-h-old blood agar plates and resuspended in 500 μL of 1 × PBS buffer, homogenized in a thermomixer at 25 °C, and shaken at 400 rpm for 2 h.

Sporulation of the strain BaML_7 was induced by cultivation in Nutrient broth (Merck, Darmstadt, Germany) for 7 days at room temperature. The spore suspension was prepared as described by Knüpfer et al. (2020).

### 2.2 Preparation of spore samples and spiked environmental samples

For the detection effectiveness assessment assays, triplicates of *B. anthracis* spore suspensions containing 10^8^ to 10^0^ spores/sample were diluted in 200 μL of 1 × PBS buffer.

The assay’s specificity was evaluated by testing samples containing spores of *B. anthracis* strain BaML_7 mixed with spores of the anthrax close relatives *B. cereus* strain ATCC 11778 and *B. thuringiensis* strain ATCC 33679, as well as spore suspensions and DNAs that did not contain *B. anthracis*. To test for specificity, we prepared duplicates of 200 μL spore samples containing 10^7^ spores of *B. cereus* and *B. thuringiensis* and 10^8^, 10^5^, and 10^4^ spores of *B. anthracis*. Additionally, we prepared duplicates of 200 μL samples containing 10^7^ CFU of *B. cereus* and *B. thuringiensis*, as well as 10^7^ CFU of either *B. cereus* or *B. thuringiensis* spores.

The performance and effectiveness of the method were tested on spiked environmental samples. Three sample types were used in experiments with spiked environmental samples: humic sandy type soil, muddy water from a puddle, and swab sample from a concrete surface. The samples were spiked with 10^8^ to 10^0^ CFU spores of the virulent *B. anthracis* strain BaML_7. Three series of all three types of samples were prepared, and a total of 90 samples were used for MLVA analysis with Nanopore sequencing. Each series consisted of triplicates of samples spiked with nine concentrations of spore suspensions and unspiked samples that did not contain *B. anthracis* spores. Prior to DNA isolation, the spores were separated from different sample matrices and concentrated to 200 μL of 1 × PBS buffer, as described below.

900 μL amounts of muddy water from a puddle were spiked with 100 μL of 10^8^ to 10^0^ CFU spore suspension. The samples were centrifuged for 10 min at 10,000 rpm, and the pellet was resuspended in 200 μL 1 × PBS buffer.

1 g amounts of soil were spiked with 100 μL of 10^8^ to 10^0^ CFU spore suspension and incubated for 30 min at room temperature. Before DNA isolation, 1 mL 1 × PBS buffer was added to the spiked soil samples, and spores were separated from the sample matrix with 2 min rigorous vortexing and centrifuging for 1 min at 1000 rpm. The spore-containing supernatants of samples were transferred to a clean microcentrifuge tube and centrifuged for 10 min at 10,000 rpm, and the pellet was resuspended in 200 μL of 1 × PBS buffer.

For each swab sample, a 10 cm × 10 cm wet and dirty concrete surface was sampled with a cotton swab (Copan, Brescia, Italy). After drying for 2 h at room temperature, swabs were spiked with 10 μL of 10^8^ to 10^0^ CFU spore suspension, and dried for 30 min. The swabs were then placed in 1 mL 1 × PBS buffer in 15 mL tubes and vortexed at high speed for 2 min. Residual liquid from the swabs was released by pressing the swabs against the inside of the tubes using sterile polypropylene forceps. The spore suspensions recovered from the swabs were transferred to clean microcentrifuge tubes and centrifuged for 10 min at 10,000 rpm. The pellet of each sample was resuspended in 200 μL of 1 × PBS buffer.

### 2.3 DNA isolation

DNA was isolated from 500 μL of homogenized vegetative bacterial suspensions of 11 virulent and one non-virulent strain, using the DNeasy Blood & Tissue Kit (Qiagen, Hilden, Germany), following the manufacturer’s protocol for Gram-positive bacteria. The concentration and purity of DNA were determined using a NanoDrop 2000C spectrophotometer (Thermo Fisher Scientific, Waltham, USA) and QuantiFluor One dsDNA System (Promega, Madison, USA). 200 μL aliquots of 1 ng/μL working solutions were prepared. The DNA stock solutions were stored at −80 °C, and the working solutions were stored at +4 °C.

DNA was isolated from 200 μL of *B. anthracis* spore suspensions and 200 μL of spore suspensions extracted from water, soil, and swab samples, starting with a mechanical disruption step described by Knüpfer et al. (2020) with some modifications. Briefly, 200 mg of 0.1 mm PowerBeads (Qiagen, Hilden, Germany) were added to each *B. anthracis* spore suspension, and the samples were lysed for 5 min at 30 Hz in a TissueLyser II instrument (Qiagen, Hilden, Germany). The beads were then sedimented by centrifugation for 1 min at 1000 rpm. DNA isolation from the supernatants was continued using the DNeasy Blood&Tissue Kit (Qiagen, Hilden, Germany), following the manufacturer’s protocol for Gram-positive bacteria. To eliminate potential PCR inhibitors, DNA isolated from the soil and surface samples was purified using a Qiagen DNeasy PowerClean Pro Cleanup Kit (Qiagen, Hilden, Germany), following the manufacturer’s protocol.

### 2.4 Quantitative real-time PCR

qPCR was used to determine the copy number of *Bacillus anthracis* DNA isolated from the spore samples. This was based on a PCR assay using the chromosomal PL3 marker described by Wielinga et al. (2011). The DNA used for the standard curve was isolated from the vegetative bacterial suspension of strain BaML_7. Standard curves were generated by sevenfold serial dilutions of purified target amplicons (PCR products containing target sequences) in the range of 10^9^ to 10^0^ copies/reaction. PCR assays were carried out in a 20 μL reaction volume containing 10 μL of Brilliant III UltraFast qPCR Master Mix (Agilent, Santa Clara, USA), 250 nM of each primer and probe, and 2 μL of target DNA. PCR reactions were performed on a CFX96 Touch thermocycler (Bio-Rad, Hercules, USA). The primer sequences and reaction parameters for the real-time PCR assay are listed in Supplementary Table S1.

### 2.5 canSNP typing

To genotype the 11 Hungarian strains, we performed canonical SNP analysis of 13 markers using the Delayed Mismatch Amplification Assay (DMAA) described by Antwerpen et al. (2019). Primer sequences were based on the modification of Melt-MAMA primers designed by Birdsell et al. (2012). We designed canSNP-DMAA primer oligonucleotides for the 13 markers using the method outlined by Antwerpen et al. (2019). Each primer pair was employed in a single-plex reaction in a 20 μL reaction volume, containing 10 μL of Brilliant III UltraFast Sybr Green qPCR Master Mix (Agilent, Santa Clara, USA), 250 nM of each primer, and 2 μL of the target DNA. The PCR reactions were performed on a CFX96 Touch thermocycler (Bio-Rad, Hercules, USA). The sequences of the canSNP-DMAA primers and the reaction conditions for real-time PCR assays are listed in Supplementary Table S1.

### 2.6 MLVA analysis with reference methods

MLVA analysis using 31 loci for the 12 *B. anthracis* strains was performed as outlined by Thierry et al. (2014), with modifications (Supplementary Table S2). A total of 31 VNTR markers were amplified in nine multiplex and eight monoplex PCRs using primers labeled with FAM or Yakima Yellow fluorescent dyes. PCR reactions were prepared using Phusion Green High-Fidelity DNA Polymerase (Thermo Fisher Scientific, Waltham, USA) as per the manufacturer’s instructions in a 20 μL PCR reaction volume with 2 μL of target DNA. PCR amplification was performed using a Veriti thermal cycler (Thermo Fisher Scientific, Waltham, USA) under the conditions listed in Supplementary Table S2. 1 μL of PCR products was separated by capillary electrophoresis on an ABI 3500 DNA analyzer (Thermo Fisher Scientific, Waltham, USA). Fragment sizes were estimated using 3,500 Series Data Collection Software (Thermo Fisher Scientific, Waltham, USA) by comparison to a GeneScan 500 LIZ Size Standard (Thermo Fisher Scientific, Waltham, USA). The copy numbers of repeating elements were calculated from the raw data of fragment lengths and were expressed as codes for repeat units. Each VNTR marker for each strain was amplified using unlabelled primers, and both strands of amplicons were sequenced using BigDye Terminator v1.1 Cycle Sequencing Kit (Thermo Fisher Scientific, Waltham, USA) and ABI 3500 DNA Analyser (Thermo Fisher Scientific, Waltham, USA). The final sequence was based on data from both strands.

### 2.7 Nanopore amplicon sequencing

A full step-by-step protocol for multiplex PCR, library preparation, and Nanopore sequencing run has been deposited in the protocols.io repository,^1^ which contains the sequences and characteristics of primers and the amplicon sizes.

Briefly, amplicons were generated from isolated DNA using Q5 Hot Start HF Polymerase (New England Biolabs, Ipswich, USA) in two parallel multiplex PCR reactions with two different primer pools (namely: BaMLVA Primer Pool 1 and Pool 2). The primers that produced PCR products shorter than 200 base pairs in the original MLVA31 typing system (CG3, pXO1, pXO2, vrrB1, vrrB2, vntr12, vntr19, vntr23 and vntr35) were redesigned to be suitable for Nanopore sequencing. The product lengths and annealing temperatures were chosen to match those of the other MLVA31 primers. Primer concentrations were optimized to ensure balanced amplification of the products in the multiplex PCR. The primers were designed using the Primer BLAST program within the NCBI.^2^

Amplicons were quantified with QuantiFluor One dsDNA System on a Quantus fluorometer (Promega, Madison, USA). Amplicons from Pool 1 and Pool 2 PCR reactions were pooled in a 1:9 ratio and diluted in 12.5 μL of nuclease-free water to a total amount of 50 ng. Library preparation was performed using the Native Barcoding Kit 96 V14 (Oxford Nanopore Technologies, Oxford, UK), following the manufacturer’s protocol for Ligation sequencing amplicons – Native Barcoding Kit 96 V14 (SQK-NBD114.96) (version: NBA_9170_v114_revQ_02Jul2025). 25 ng of final libraries were loaded onto an R10.4.1 (FLO-MIN114D) flow cell (Oxford Nanopore Technologies, Oxford, UK) and sequenced for 15 h for pure spore dilutions and spiked samples and for 24 h for the 12 different strains, the non-*B. anthracis B. cereus* strains and the mixed samples, using an ONT GridION device (Oxford Nanopore Technologies, Oxford, UK).

### 2.8 Bioinformatics

Raw ONT signal data were basecalled using “superaccurate basecalling” mode, de-multiplexed, filtered out reads with quality score less than Q12 and trimmed in real-time during sequencing run using default parameters within the dorado workflow in MinKNOW (Release 24.11.8) installed on GridION device (Oxford Nanopore Technologies, Oxford, UK). The version 7.6.7. of dorado-basecall-server-for-gridion included in the used MinKNOW release 24.11.8 matches the dorado version 0.7.4.

We developed a pipeline named Bant_MLVA31_analyzer, which determines the repeat numbers for the 31 *B. anthracis* VNTR loci from basecalled and de-multiplexed raw reads, generates consensus sequences of repeat regions, and checks the similarity of the generated sequences to *B. anthracis*. The pipeline is publicly available at GitHub.^3^ The steps of the pipeline are summarized below.

The corresponding passed fastq files containing trimmed and quality-filtered reads for each barcode were merged, renamed to the sample name, and one fastq file per sample was used as input into the Bant_MLVA31_analyzer pipeline. Each fastq file was aligned to the *Bacillus anthracis* ‘Ames Ancestor’ reference genome (GCF_000008445.1) using minimap2 (v2.17), and the reads were split into 31 fastq files per sample by the repeat regions using samtools (v1.10). Reads were then filtered by aligning to a collection of reference sequences of each repeat region, and reads mapped to a given reference sequence with a value of maximum mean depth of coverage were exported with samtools (v1.10). The exported reads served as input to EMBOSS primersearch (v6.6.0.0) for *in silico* PCR to generate amplicons for 31 VNTR regions with primers from the MLVA31 typing scheme using the -mismatchpercent 10 option. The reads are filtered further by the distribution of the length of amplicons from *in silico* PCR. The descriptive statistics of the length distribution of the amplicons were calculated using R (v4.2.1) with an in-house script, and the reads containing amplicon lengths between the minimum and maximum values were exported using seqtk (v1.3). The exported reads were used to determine the length of the VNTR marker, thereby defining the number of exported reads as the number of useful reads that is the number of reads used for the analysis. If the number of exported reads was greater than 20, a consensus sequence was generated using the amplicon_sorter tool (Vierstraete and Braeckman, 2022). The consensus sequence was aligned to the database of *Bacillus cereus* group reference genomes^4^ using BLASTN (v2.10.1+) to determine the percentage of identity with *B. anthracis* sequences. If the sequence with the highest percentage of identical matches is *B. anthracis*, the exact length of each of the 31 repeat regions is determined by the consensus sequence. If the result of BLASTN was not *B. anthracis* or the number of reads from the previous step was less than 20, the raw reads exported with seqtk were aligned one by one to the *Bacillus cereus* group genome database with BLASTN (v2.10.1+). If more than 90% of the reads were matched to *B. anthracis* with the highest percentage of identical matches, the exact length of repeat regions was calculated using the value of median from the descriptive statistics of the length distribution of the amplicons generated by *in silico* PCR. If the raw reads had not yield *B. anthracis* as a result of BLASTN analysis, the length of the repeat regions could not be ascertained. In the final step of the pipeline, the number of repeat units was calculated from the length of the repeats according to the coding convention of Thierry et al. (2014) using R (v4.2.1) with an in-house script. The results were exported to a CSV table for each sample.

The pipeline includes a module that compares the MLVA profile of the analyzed strain with a global *Bacillus anthracis* MLVA database and determines the strains that are the closest neighbors to it using an in-house R script. This is described in detail in the following section.

### 2.9 Comparison to public databases

To compare the MLVA genotypes of the Hungarian strains with data from other countries, we created a comprehensive in-house *Bacillus anthracis* MLVA database with MLVA profiles of 559 strains with known geographic origins. The database was deposited at Zenodo data repository.^5^ The data of strains have been obtained from the public *Bacillus anthracis* database (MLVABank for Microbes Genotyping, 2023) and from previously published sources.

We developed an in-house R script for the identification of the nearest neighbors of a particular *B. anthracis* strain using the global *B. anthracis* MLVA database. First, a pairwise dissimilarity matrix of the strains was constructed from the repeat number data of the identified VNTR markers (variables) using the following repeat-dissimilarity coefficient:


$$D_{\mathrm{ij}}=1-A/(A+B)$$

where *Dij* is the value of repeat-dissimilarity between strains *i* and *j*; *A* is the frequency of matching cases or variables (markers); and *B* is the frequency of non-matching cases (markers). *Dij* ranges from 0 (total similarity) to 1 (total dissimilarity). We considered a case (marker) matching between *i* and *j* if the marker was present in both strains and the repeat numbers of the marker were identical (0,1,2,…, etc.) in the two strains. If the marker was present in both *i* and *j* strains and the repeat numbers were not identical, the case was considered non-matching. If a marker was not present in one of the strains or in both, it was excluded from the comparison. Therefore, *A* + *B* equals the number of markers present in both *i* and *j* strains. Second, the identification of the nearest first, second, third, and so on, neighbors of a particular strain was achieved simply by sorting the *D_ij_* repeat-dissimilarities in ascending order. Finally, an agglomerative hierarchical cluster analysis with an UPGMA algorithm (Unweighted Pair Group Method with Arithmetic means) of the *Dij* dissimilarity matrix was also conducted to illustrate graphically the genotype relationships between the strains.

MLVA profiles of the 11 Hungarian strains and the 559 strains in the in-house MLVA database served as input for the construction of a minimum spanning tree using GrapeTree v1.5.0 (Zhou et al., 2018).

## 3 Results

### 3.1 Performance evaluation of the method on 12 *Bacillus anthracis* strains

The performance of the novel method was evaluated using two outcomes of analysis: the number of correctly identified repeats and the number of consensus sequences identical to the reference. In all 12 strains, the repeat numbers of all 31 loci were correctly determined and showed complete agreement with the reference data (Supplementary Table S3).

The consensus sequences for all 31 repeat regions were found to be 100% accurate for all strains compared to the result of Sanger sequencing. The sequencing statistics for the amplicon-based NGS assays performed using the 12 *B. anthracis* strains are shown in Supplementary Table S3. As the length of the PCR amplicons varied between 212 and 952 bases, depending on the strain, the average read length ranged from 380.2 to 447.7 bases across the different isolates. The median Q-scores of the reads ranged from 22.7 to 23.5. Due to the multiple filtering steps of the bioinformatic pipeline, the percentage of reads used for the final determination of repeat numbers was 45–55% of the total number of sequencing reads.

### 3.2 Assessment of the limit of detection of the novel method using pure spore dilution

The test of the tenfold serial dilution of the BaML_7 pure spore suspension showed, that repeat numbers of all VNTR loci were correctly identified from all three samples with 10^8^ to 10^4^ CFU/sample spore concentration (Table 1; Supplementary Tables S4, S5). Consensus sequences for all 31 loci were successfully generated from all three samples with 10^8^ CFU spores. However, one sample with 10^7^, two samples with 10^6^, and one sample with 10^4^ CFU/sample spore concentration resulted in 31 consensus sequences. For samples with a spore concentration of 10^3^ CFU/sample, the correct number of 29–30 repeats were identified. From samples with 10^2^ and 10^1^ CFU spores 24–30 VNTR loci were successfully identified. Samples with the lowest 10^0^ CFU/sample spore concentration resulted in the correct identification of 12–17 loci. We defined the limit of detection (LoD) of the method as the lowest spore concentration at which all 31 VNTRs were identified in all replicates. In accordance with this definition, the LoD of our novel method was determined at 10^4^ CFU/sample spore concentration for pure spore suspension samples.

**Table 1:** DNA concentrations, number of generated sequencing reads used in MLVA analysis, and number of MLVA loci with correct repeat numbers in different sample types with 10^8^ to 10^0^ CFU/sample *Bacillus anthracis* spore concentration.

Spore concentration (CFU spore/sample)	Sample type	DNA concentration (copies/reaction)			Number of useful sequencing reads			Number of correctly identified repeats		
		1	2	3	1	2	3	1	2	3
1.00E+08	Spore suspension	2.6E+07	9.0E+06	5.4E+06	1.1E+06	5.9E+05	4.2E+05	31	31	31
	Muddy water	1.3E+07	7.2E+06	2.5E+06	1.4E+06	8.2E+05	2.6E+05	31	31	31
	Soil	6.5E+05	6.3E+05	4.6E+05	2.4E+05	1.7E+05	1.1E+05	31	31	31
	Swab	1.3E+06	3.6E+05	3.0E+05	2.0E+05	1.3E+05	4.7E+04	31	31	31
1.00E+07	Spore suspension	3.4E+06	1.3E+06	5.5E+05	2.2E+05	7.9E+04	9.3E+04	31	31	31
	Muddy water	1.8E+06	1.1E+06	5.8E+05	2.0E+05	6.9E+04	1.1E+05	31	31	31
	Soil	8.9E+04	8.0E+04	5.8E+04	1.0E+05	3.9E+04	2.1E+04	31	31	31
	Swab	9.6E+04	6.1E+04	5.3E+04	8.9E+04	1.2E+05	3.5E+04	31	31	31
1.00E+06	Spore suspension	3.2E+05	2.5E+05	1.7E+05	4.8E+04	4.6E+04	3.4E+04	31	31	31
	Muddy water	1.9E+05	1.8E+05	6.4E+04	4.9E+04	7.1E+04	6.4E+04	31	31	31
	Soil	5.2E+03	4.3E+03	4.1E+03	3.1E+04	2.1E+04	1.7E+04	31	31	31
	Swab	9.1E+03	4.8E+03	4.1E+03	5.9E+04	2.1E+04	1.9E+04	31	31	31
1.00E+05	Spore suspension	6.0E+04	5.0E+04	2.9E+04	6.3E+04	4.6E+04	4.3E+04	31	31	31
	Muddy water	1.8E+04	1.8E+04	3.3E+03	4.7E+04	5.0E+04	1.7E+04	31	30	30
	Soil	9.3E+02	4.8E+02	4.7E+02	1.3E+04	6.2E+03	4.4E+03	30	29	28
	Swab	9.8E+02	4.1E+02	2.3E+02	1.4E+04	6.2E+03	2.0E+03	31	25	24
1.00E+04	Spore suspension	1.7E+04	5.8E+03	4.9E+03	4.4E+04	2.5E+04	2.6E+04	31	31	31
	Muddy water	2.7E+03	1.6E+03	9.0E+02	1.7E+04	6.6E+03	1.8E+04	29	27	28
	Soil	3.2E+02	3.0E+02	1.9E+02	1.3E+03	2.4E+03	6.9E+02	25	23	20
	Swab	1.8E+02	1.3E+02	4.1E+01	9.5E+02	6.9E+02	3.4E+02	22	15	17
1.00E+03	Spore suspension	3.9E+03	2.6E+03	1.6E+03	2.4E+04	1.3E+04	5.1E+03	30	30	29
	Muddy water	8.7E+02	7.6E+02	6.1E+02	9.3E+03	2.7E+03	3.8E+03	27	27	25
	Soil	1.7E+02	1.4E+02	7.9E+01	8.6E+02	6.9E+02	4.9E+02	22	18	17
	Swab	3.2E+01	2.9E+01	2.0E+01	3.3E+02	2.6E+02	4.9E+02	17	16	13
1.00E+02	Spore suspension	7.4E+02	5.8E+02	4.4E+02	7.3E+03	5.6E+03	1.2E+03	30	27	24
	Muddy water	5.0E+02	4.9E+02	4.3E+02	4.2E+02	1.4E+03	1.1E+03	17	26	24
	Soil	4.2E+01	4.0E+01	3.3E+01	3.0E+02	2.8E+02	2.5E+02	18	15	12
	Swab	1.2E+01	0.0E+00	0.0E+00	2.5E+02	0.0E+00	0.0E+00	12	0	0
1.00E+01	Spore suspension	4.0E+02	3.6E+02	3.2E+02	4.4E+03	4.0E+03	1.6E+03	27	29	24
	Muddy water	2.8E+02	2.4E+02	1.8E+02	7.3E+02	1.0E+03	6.0E+02	19	27	20
	Soil	3.0E+01	2.7E+01	2.7E+01	1.7E+02	2.9E+02	2.9E+02	13	12	13
	Swab	0.0E+00	0.0E+00	0.0E+00	0.0E+00	0.0E+00	0.0E+00	0	0	0
1.00E+00	Spore suspension	1.9E+02	9.1E+01	6.1E+01	4.4E+02	2.1E+02	2.8E+02	17	13	12
	Muddy water	1.2E+02	9.6E+01	5.1E+01	2.5E+02	2.7E+02	2.4E+02	12	10	11
	Soil	1.3E+01	8.8E+00	0.0E+00	2.0E+02	1.4E+02	0.0E+00	12	11	0
	Swab	0.0E+00	0.0E+00	0.0E+00	0.0E+00	0.0E+00	0.0E+00	0	0	0

Data of samples with 31 identified VNTR loci are marked in green, and data of samples with no identified repeats are marked in yellow.

To further analyze the performance of the method with regard to the detection effectiveness of samples with varying spore concentrations, we examined the copy number of isolated DNA, the number of total and useful sequencing reads per sample, and the sequencing depth (Table 1; Supplementary Tables S4, S5). The copy number of DNA in the samples at 10^8^ to 10^4^ CFU/sample spore concentration, which resulted in the correct identification of all 31 repeats, was above 4.9 × 10^3^ copies/reaction. The minimum number of useful sequencing reads that resulted in the correct identification of all 31 VNTRs was 2.4 × 10^4^. In the samples with 31 identified VNTRs an average of 48% of the total raw reads was used for analysis. Consequently, the minimum number of total sequencing reads that resulted the correct identification of all the 31 VNTRs was 5.1 × 10^4^ (sample 10^4^/3). The mean sequencing depth for samples containing 10^4^ CFU/sample spore was found to range from 1,289x to 2,284x, with variations observed between 23x and 12,046x across the three replicates for different VNTR loci (Supplementary Table S5). The lowest sequencing depth was observed in the bams22 and vntr16 loci (23x – 668x and 23x – 419x, respectively), and the highest in the vntr19 and bams28 loci (7,725x – 10,937x and 4,045x – 12,046x, respectively).

In samples with a spore concentration of less than 10^4^ CFU/sample, a decline in the number of correctly identified repeats and successfully generated consensus sequences was observed, which is attributable to a decrease in DNA concentration and sequencing reads. In addition, the percentage of useful reads from the total raw reads in these samples decreased to an average of 29% (Supplementary Table S4). Due to the lack of generated sequencing reads, the bams22, bams30 and vntr16 loci could not be identified from samples at 10^3^ CFU spores. In case of bams22 the repeat number was successfully determined from 30x sequencing depth, and in vntr16 from 9x, however in case of bams30 the required depth was minimum 52x. The lowest DNA copy number that resulted in the identification of any repeats was 61 copies/reaction in the sample with a spore concentration of 10^0^ CFU/sample. The lowest number of useful sequencing reads was 209, obtained from a total of 1 × 10^3^ raw reads, which resulted in the identification of 13 repeats, and two consensus sequences were generated.

The median quality score of the sequencing reads ranged from 20.3 to 23.

### 3.3 Evaluation of the performance and assessment of the limit of detection of the novel method using spiked samples

The test of the spiked environmental samples showed that all 31 VNTRs were identified in all replicates of all sample types spiked with 10^8^ to 10^6^ CFU of *B. anthracis* spores (Table 1; Supplementary Tables S4, S6–S8). Consequently, the limit of detection (LoD) of our novel method was determined at 10^6^ CFU/sample for all types of spiked environmental samples. In samples with a spore concentration of 10^5^ CFU/sample, muddy water samples yielded the highest number of identified loci (30–31), whereas the swab samples had the lowest (24, 25, and 31). However, one swab sample at this spore concentration resulted in the correct identification of all 31 loci. A similar trend was observed in samples containing 10^4^ CFU spores or fewer, with muddy water samples exhibiting the highest average number of correctly identified loci and swab samples exhibiting the lowest. Two of the three replicates of swab samples at 10^2^ CFU, and all swab samples at 10^1^ and 10^0^ CFU spores did not contain detectable DNA, and none of the 31 VNTRs were identified. The environmental samples were sequenced without spiking with *B. anthracis* spores, and no sequencing reads specific to any of the 31 VNTRs analyzed were generated.

As the quantity of DNA copies decreased, a corresponding decline in the number of sequencing reads was observed (Table 1; Supplementary Tables S4, S6–S8). The DNA concentration and number of total raw sequencing reads as well as the number of useful reads in muddy water were one magnitude higher than in the soil and swab samples at all samples with higher than 10^1^ CFU/sample spore concentrations and were roughly equal to that of the pure spore samples. The DNA concentration and read number of soil and swab samples were consistent in samples containing 10^8^ to 10^5^ CFU spores. However, at 10^4^ and 10^3^ CFU spores, the number of DNA copies and sequencing reads obtained from the swab samples decreased to approximately 50–20% of that acquired from the soil samples. The lowest DNA copy number from which all 31 VNTRs were successfully identified was 1.8 × 10^4^ in muddy water samples, 4.1 × 10^3^ in soil samples, and 9.8 × 10^2^ in swab samples.

The number of useful sequencing reads in these samples were 4.7 × 10^4^, 1.7 × 10^4^, and 1.4 × 10^4^, respectively. This useful read number was gained from 1.2 × 10^5^ total raw reads in muddy water, 3.6 × 10^4^ in soil and 4.9 × 10^4^ in swab samples. However, the highest number of useful reads from which only 30 VNTRs were identified was 5.0 × 10^4^ from a muddy water sample with 1.8 × 10^4^ DNA copies. These results show that a minimum of 2.0 × 10^4^ to 5.0 × 10^4^ useful sequencing reads are required for the successful identification of all 31 VNTRs from spiked samples, with a minimum of 4.0 × 10^4^ to 1.2 × 10^5^ total raw reads necessary. These results are comparable to those observed from pure spore samples.

With regard to the ratio of the used reads to the total raw reads in the spiked samples, the result is comparable to those obtained from pure spore samples. This implies that of the muddy water, soil and swab samples from which all 31 VNTRs were successfully identified, an average of 45, 42, and 47% of total raw reads were used for analysis, respectively. The minimum sequencing depth required for identification of all VNTR loci in spiked samples was 1,041x in soil, 1,295x in swab samples, and 2,412x in muddy water samples. In summary, it was observed that the DNA copy number, total and useful read number, and sequencing depth required for successful identification of all 31 VNTRs was 2–5x higher in the muddy water samples than in the soil, swab, and pure spore samples.

The median quality score of the sequencing reads in the spiked samples ranged from 19.8 to 23, which is comparable to the Q-scores of the pure spore samples.

### 3.4 Evaluation of specificity using mixed samples of spores

**Figure 2:**
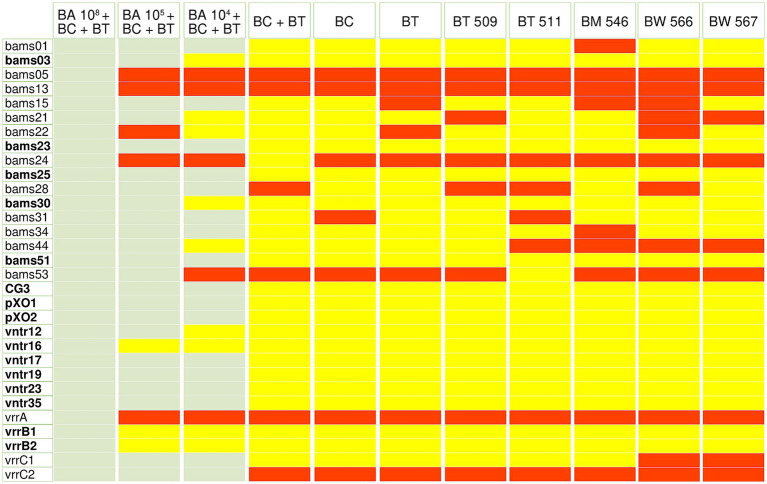
Results of the *in silico* MLVA analysis of the amplicon-based NGS data generated from the mixed spore samples and non-*Bacillus anthracis Bacillus cereus*-group species containing samples. *Bacillus* species and *Bacillus anthracis* spore concentrations as CFU/sample mixed with samples are displayed across the top. BA, *Bacillus anthracis* BaML_7; BC, *Bacillus cereus* ATCC 11778; BT, *Bacillus thuringiensis* ATCC 33679; BT 509, *Bacillus thuringiensis* B509; BT 511, *Bacillus thuringiensis* B511; BM 546, *Bacillus mycoides* B546; BW 566, *Bacillus weihenstephanensis* B566; BW 567, *Bacillus weihenstephanensis* B567. 31 VNTR markers are displayed in the left side. Green boxes indicate markers with correct repeat numbers, yellow boxes indicate that the repeat number was not determined due to lack of sequencing reads, and red boxes indicate incorrect repeat numbers that are not specific for *Bacillus anthracis*. The VNTR loci written in bold did not produce PCR products with the primers used, and no sequencing reads were obtained for samples containing no *Bacillus anthracis*. One of the two replicates of each sample is shown in the figure.

The specificity test using mixed spore samples showed, that in samples where the concentration of *B. anthracis* spores was higher than that of other closely related *Bacillus* species, the number of all repeats was correctly determined (Figure 2; Supplementary Table S9). In samples containing 10^5^ and 10^4^ CFU/sample of *B. anthracis* spores mixed with higher amounts of spores of other *Bacillus* species, five to six repeats (bams05, bams13, bams22, bams24, bams53, vrrA, and vrrC2, marked in red in Figure 2; Supplementary Table S9) in each sample produced high numbers of sequencing reads. However, these reads were generated from non-*B. anthracis* specific PCR products, and the correct number of repeats could not be determined, resulting in false negative results. The correct number of repeats for 23, 21, and 17 VNTR loci were determined from samples containing 10^5^ and 10^4^ CFU/sample of *B. anthracis* spores mixed with other *Bacillus* species.

Spore suspensions containing *B. cereus* and *B. thuringiensis,* or only *B. cereus or B. thuringiensis,* and DNA from 5 different *B. cereus* group species was used to further test the performance of the method on *Bacillus* species closely related to *B. anthracis*. In samples containing only non-*B. anthracis* species, a total of 15 loci generated sequencing reads and consensus sequences. However, these loci did not provide repeat numbers because the consensus sequence was not specific to *B. anthracis*. Six of these regions were the same as those that gave false-negative results in samples containing 10^5^ and 10^4^ CFU/sample of *B. anthracis* spores (bams05, bams13, bams22, bams24, bams53, vrrA, and vrrC2). The results suggest that these repeat regions are present in the genomes of *B.cereus* group species closely related to anthrax because of the high genetic similarity within the *B. cereus* group. In our assays, 16 regions did not produce PCR products with the primers used, and no sequencing reads were obtained in samples containing no *B. anthracis* (bams03, bams23, bams25, bams30, bams51, CG3, pXO1, pXO2, vntr12, vntr16, vntr17, vntr19, vntr23, vntr35, vrrB1, and vrrB2, written in bold in Figure 2). This indicates that these loci are specific to *B. anthracis*.

### 3.5 Genotypes of the wild Hungarian strains

**Figure 3:**
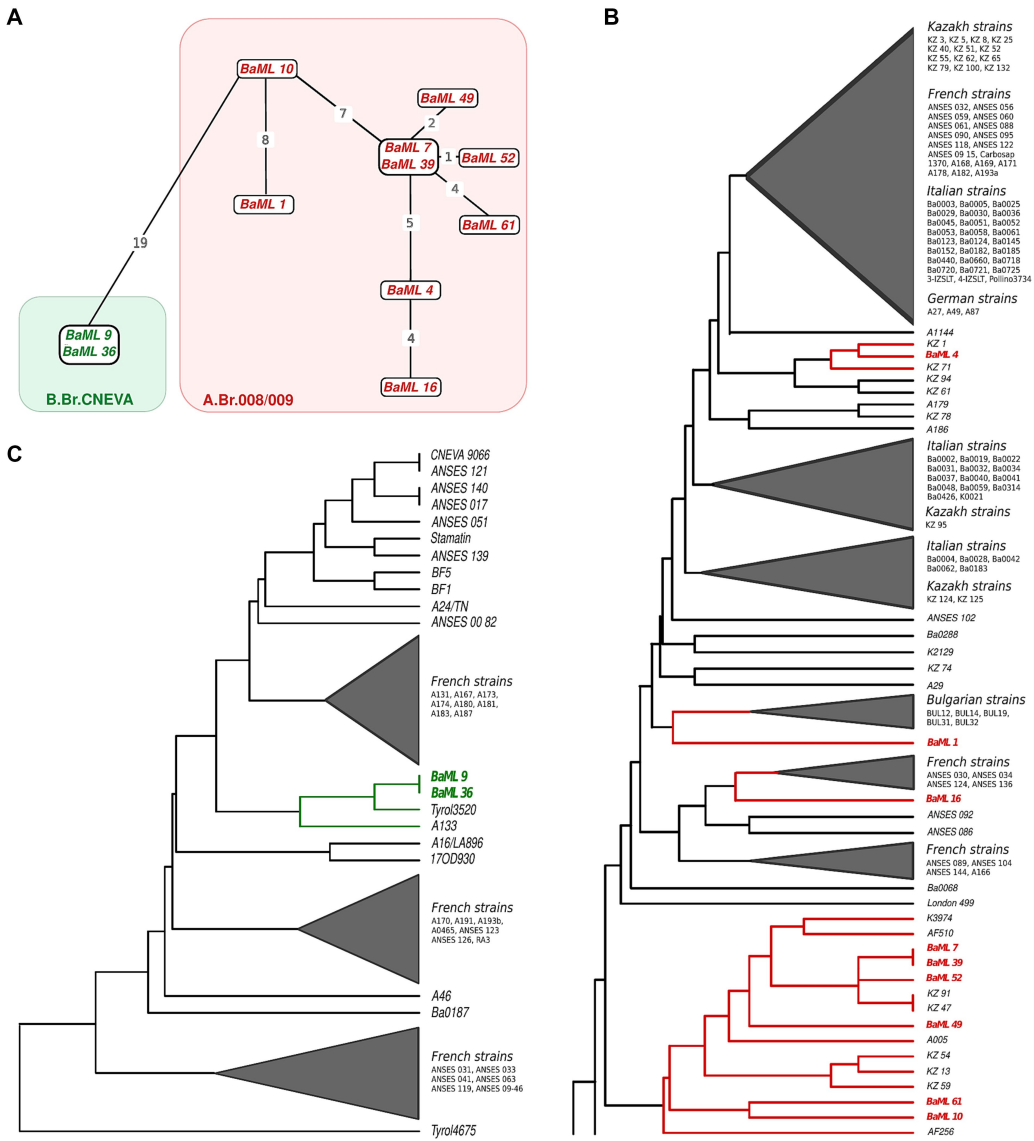
Phylogenetic analysis of 11 Hungarian *Bacillus anthracis* strains. **(A)** Minimum spanning tree calculated by GrapeTree using MLVA genotypes relied on the chromosomal markers of the 11 Hungarian *Bacillus anthracis* strains and canSNP genotypes of the isolates. Two strains from the B.Br.CNEVA clade are marked in green, and nine strains marked in red belong to the A.Br.008/009 clade. **(B)** The position of nine Hungarian A.Br.008/009 strains in the UPGMA cluster analysis of 559 distinct genotype *Bacillus anthracis* isolates based on 27 chromosomal MLVA markers. The UPGMA cluster shows the representative part of clade A, showing the closest MLVA relatives of the Hungarian strains. **(C)** The position of two Hungarian B.Br.CNEVA strains in the UPGMA cluster analysis of 559 distinct genotype *Bacillus anthracis* isolates based on 27 chromosomal MLVA markers is shown in detail within the isolates belonging to the B2 branch.

Genotyping of 13 canSNP markers separated the 11 wild-type Hungarian isolates into two phylogenetic groups. Nine of the 11 strains belong to the A.Br.008/009 Trans-Eurasian (TEA) group within clade A, whereas two strains belong to the B.Br.CNEVA group of clade B (Figure 3A). In the determination of closest neighbors and cluster analysis of MLVA genotypes, we only compared the MLVA genotypes of Hungarian strains with those belonging to the same canSNP group. We did not include markers on plasmids (pXO1, pXO2, vntr16, vntr17) in the analysis to avoid false clustering of plasmid-free strains. The 11 wild Hungarian strains show nine different genotypes identified by the 31-marker MLVA. From nine strains from the A.Br.008/009 canSNP group four strains show very close genetic subtypes (Figures 3A,B; Supplementary Figure S1), and BaML_7 and BaML_39 have identical MLVA patterns, with only one and two allele differences from the strains BaML_49 and BaML_52. This group comprises the Hungarian BaML_61 and BaML_10 strains, which differ by three chromosomal alleles from each other and four and seven alleles from strains BaML_39 and BaML_7. The closest relatives of these isolates are strain A005 from Austria, strains KZ_47 and KZ_91 from Kazakhstan and strain K3974 from Slovakia with 1–3 allelic differences.

The strains BaML_16, BaML_4 and BaML_1 have distinct MLVA genotypes, differing from the BaML_7 and BaML_39 strains with eight, seven and 11 alleles, respectively. These strains clustered at three different branches within a sister group of the subclade containing the aforementioned six Hungarian strains. The BaML_1 strain has been shown to be closely related to a group of Bulgarian strains (BUL12, BUL14, BUL19, BUL31 and BUL32), with a distance of three to four alleles. The strain BaML_16 clustered together with French strains (ANSES030, ANSES034, ANSES124, ANSES136), having 2–4 allele difference. The closest relatives of the BaML_4 strain are the Kazakh strains with a genetic distance of one allele from KZ_1, two alleles from KZ_5, KZ_61 and KZ_71, and three alleles from KZ_94.

The BaML_9 and BaML_36 strains belong to the B.Br.CNEVA subclade (Figure 3A). The two strains have identical repeat numbers in chromosomal VNTR markers. However, these two strains are plasmid-less, as the BaML_9 lacks both plasmids and the BaML_36 lacks plasmid pXO1. The Hungarian B.Br.CNEVA isolates clustered together with the Austrian Tyrol 3520 isolate, having one chromosomal allele difference and this group includes the German A133 isolate (Figure 3C). The closest relatives of these strains from the European B branch are isolated in France and Germany as well as the Slovakian A24/TN_Bovine_Sokol strain.

## 4 Discussion

The precise strain-level genetic identification of an unknown *Bacillus anthracis* strain directly from environmental samples contributes to a timely and accurate response to biological events caused by natural outbreaks or bioattacks. The on-site rapid molecular subtyping of *B. anthracis* strains facilitates the assessment of whether the strains are representative of the naturally established strains within the region or they have been imported from other areas, including laboratory culture collections. The optimal rapid genetic subtyping method for the aforementioned purposes (1) should be high resolution enough to provide precise differentiation between two *B. anthracis* isolates; (2) should enable the differentiation of *B. anthracis* from the close relative and genetically highly similar *B. cereus* group species; (3) should be sensitive enough to detect low quantities of target DNA within the total amount of DNA isolated from environmental samples; (4) should be culture-free in order to ensure an appropriate speed of analysis via direct genotyping of the pathogen strain from environmental samples; (5) the results should be comparable with the genotype of *B. anthracis* strains isolated worldwide, in order to determine the origin of an unknown strain with the greatest possible accuracy; (6) should be field-applicable by portable biodefense or epidemiological laboratories.

To overcome these challenges, we developed a multiplex PCR-based amplicon sequencing assay with an ONT MinION device and an *in silico* VNTR analysis method for genotyping 31 MLVA markers of *B. anthracis* directly from environmental samples. This culture-free genotyping method provides results within one day using DNA isolated from the original environmental samples, whereas the reference capillary electrophoresis method requires approximately five to seven days to obtain pure isolates of the unknown strain prior to genotyping. Our newly developed amplicon-sequencing approach uses PCR primers from the MLVA 31 typing scheme (Beyer et al., 2012), thereby ensuring the comparability of the results with the global *B. anthracis* genotyping datasets generated using the reference capillary electrophoresis method. The custom bioinformatic pipeline compares the unknown *B. anthracis* strain with an in-house comprehensive database of 559 distinct MLVA genotypes of globally isolated *B. anthracis* strains to determine the closest relatives.

The MLVA system has been shown to have high resolution and to provide accurate information about the geographic origin of an unknown *B. anthracis* strain. Keim et al. (2000) observed a limited number of mutations in VNTR markers across multiple passages of *B. anthracis* strains in laboratory experiments, suggesting the stability of MLVA alleles of *B. anthracis* isolates over time. VNTR mutation rates have been found to be within the range of 10^−5^–10^−4^, and mutations occur in single-repeat steps (Keim et al., 2000; Keim et al., 2004; Keim et al., 2009). Therefore, it is highly improbable that distinct genotypes emerge during a single outbreak. Furthermore, a strain endemic to a given area is likely to have a unique MLVA genotype. In a recent study, 26 distinct strains were isolated from various sources (humans, animals, and soil) in four provinces of Vietnam (Metrailer et al., 2023). Applying the MLVA25 method, 11 unique genotypes were identified, which were found to be distinct from all other global MLVA genotypes. The analysis of the 559 strains included in the in-house MLVA database established in our study indicates that strains endemic to a specific region exhibit a notable degree of similarity in their genotypes and are classified into a common branch with strains originating from neighboring regions (see Supplementary Figure S1).

The MLVA typing system does not provide sufficient resolution for the individualisation of isolates in outbreaks or bioforensic investigations, what can only be achieved by wgSNP typing. Furthermore, as it is prone to homoplasy, MLVA typing is not suitable for detailed phylogenetic analysis. However, it can be used for the preliminary genetic characterization of unknown strains and can therefore provide valuable leads for further in-depth genetic analysis (Rasko et al., 2011). After the 2001 bioterrorism-associated anthrax outbreak, MLVA was used to subtype isolates from patients, the environment, and powders, and provided information about potential sources (Rasko et al., 2011). The MLVA genotype of the outbreak *B. anthracis* strain was shown to be identical to the Ames strain, which is not typically found in nature but is commonly used in research laboratories worldwide. Nevertheless, the identification of unique genetic markers providing a molecular fingerprint that cannot be found in any spore preparation other than the one from which the spores were mailed in 2001 was only possible with whole-genome sequencing of the isolates and comparative analysis using the Ames Ancestor reference genome. This supported the FBI’s investigative efforts to identify the source of the 2001 letter spores.

A major challenge in the identification and genotyping of *B. anthracis* spores in environmental samples is the presence of non-virulent *Bacillus cereus* group species, which are closely related to *B. anthracis*. MLVA was considered an unsuitable typing method for the analysis of environmental samples that may contain other members of the *B. cereus sensu lato* group because of the high level of genetic similarity between the members of this group (Turingan et al., 2013). However, in previous studies (Keim et al., 2000; Marston et al., 2006), it was observed that from the examined eight markers, close relatives of *B. anthracis*, such as *B. cereus* and *B. thuringiensis*, only amplified 0–2 VNTR loci, and any resulting allele sizes were found to differ from those observed in *B. anthracis*. Our results demonstrated that pure non-*B. anthracis B. cereus* group isolates exhibited amplification of 15 VNTR loci from the analysed 31 markers. Nevertheless, the number of repeats in these strains was distinct from those observed in *B. anthracis* strains, and the sequences of the amplified regions were different from those of *B. anthracis* and similar to those of other *B. cereus* group species. These results indicate that VNTR markers may be suitable for the unequivocal and specific identification of *B. anthracis* strains.

The limit of detection (LoD) of our method was considered as the lowest spore concentration at which all 31 VNTRs were identified in all replicates. We expressed the LoD of the novel method with three values. The analytical LoD is indicative of the method from pure spore samples. The post-isolation analytical LoD, expressed in DNA copy numbers, demonstrates the sensitivity of multiplex PCR and NGS. Finally, the matrix-specific LoD involves the effect of different sample matrices on assay performance in determining sensitivity.

The analytical LoD was found to be 10^4^ CFU/sample spore concentration in pure spore suspension, which is comparable to the sensitivity of a recent 14-plex PCR-based Nanopore amplicon sequencing method developed by Player et al. (2020) for the identification of *B. anthracis* spores. The matrix-specified LoD was identified as 10^6^ CFU/sample spore concentration. In samples with lower concentrations, the number of detectable markers was lower in environmental samples than in pure spore samples with the same spore concentration. The highest number of markers was identified in muddy water samples and the lowest in swab samples. These results indicate a correlation between the number of VNTR loci identified in environmental samples and the characteristics of the sample matrix. This can be attributed to the fact that the sensitivity of DNA-based detection, identification, and genotyping assays is influenced by the efficiency of nucleic acid extraction, which is greatly affected by the composition of the sample matrix (Dauphin et al., 2009). A previous study demonstrated that the quantity of DNA isolated from a defined concentration of a pure spore suspension was approximately 2–3 times greater than that obtained from a swab and powder sample spiked with a spore concentration identical to that of the pure suspension, using the same nucleic acid isolation kit (Dauphin et al., 2009). Therefore, to exclude the effect of the sample matrix, we examined the post-isolation analytical LoD of the multiplex PCR and NGS performed on the isolated and purified DNA. The post-isolation analytical LoD of our novel method was found to be approximately 5 × 10^3^ DNA copies/reaction in pure spore suspension and soil samples, and 10^3^ copies/reaction in swab samples. These results are comparable to those of a 52-plex PCR-based amplicon sequencing method, which was developed to detect 34 bacterial species of public health and bioterrorism significance in a recent study (Dias et al., 2026). However, in the case of muddy water samples, the post-isolation analytical LoD was found to be five times higher (1.8 × 10^4^ copies/reaction) than in pure spore, soil, and swab samples. The DNA concentrations of muddy water samples with 10^5^ CFU/sample or fewer were approximately equivalent to those of the pure spore samples. However, a reduced number of sequencing reads and a lower number of VNTRs were identified compared to those from pure spore samples. The findings indicate that the efficacy of multiplex PCR and sequencing library preparation was reduced in muddy water samples compared to other samples. The results suggest that inhibitors remained in the muddy water DNA samples, thereby inhibiting the enzyme used for multiplex PCR for sequencing but not the polymerase applied for qPCR.

In this study, we genotyped 11 Hungarian wild isolates using 13 canSNP and 31 MLVA markers. There is limited publicly accessible information on the detailed genotypes of *B. anthracis* strains isolated in Hungary. Kreizinger et al. (2016) genotyped 29 strains from a historical strain collection isolated from diverse host species in various parts of Hungary between 1933 and 2014. The 11 *B. anthracis* strains genotyped in our study are isolates from 29 previously analyzed strains. The results of the canSNP analysis were consistent with those of the previous study, as nine isolates were classified into the A.Br.008/009 TEA group and two strains into the B.Br.CNEVA group. The 11 wild Hungarian strains, classified using the MLVA31 typing scheme, represent nine distinct genotypes that are unique and differ from those represented in the global *B. anthracis* database. The Hungarian strains were divided into five groups based on their MLVA genotypes. Of these, four groups were identified as belonging to clade A, while the remaining group fell into clade B. Within clade A, the largest group comprised six strains, two of which (BaML_7 and BaML_39) were identical. The six strains were distinguished solely by 1–7 alleles, and clustered together with strains isolated from neighboring countries. These results suggest that these strains are members of the dominant endemic *B. anthracis* population in Hungary. The two Hungarian B strains formed a unique subgroup together with the Austrian Tyrol 3520 and German A133 strains within the European B branch, which includes the lineage leading to the French, German and Slovakian B.Br.CNEVA strains. The B.Br.CNEVA clade is found mainly in Western and Central Europe (Van Ert et al., 2007), with strains of this group have been isolated as autochthonous in mountainous areas of France, Germany, Switzerland, Northern Italy, Bosnia-Herzegovina, Croatia, Slovenia, Slovakia, Poland and Austria (Derzelle et al., 2016; Braun et al., 2022). A detailed phylogenetic analysis of strains belonging to the B.Br.CNEVA clade concluded that the strains of this branch were most likely introduced into the various Central European regions by a limited number of events (Braun et al., 2022), subsequently followed by local diversification of strains. This suggests *in situ* differentiation of these strains within Hungary as well, indicating a prolonged stable presence of this lineage in Hungary. A more precise phylogenetic investigation utilizing the full resolution provided by whole genome sequence (WGS), will facilitate a deeper understanding of phylogenetic relationships and the placement of the Hungarian *B. anthracis* isolates within the global *B. anthracis* population.

The limitations of our novel method presented in this study are attributable to the homoplastic nature exhibited by the VNTR loci. Interpretation of only MLVA-based typing results regarding phylogeny has to be performed with caution (Antwerpen et al., 2011). The phylogenetic relationships of *B. anthracis* strains based purely on MLVA data may be erroneous due to homoplasy that can result from rapidly evolving VNTR loci as a consequence of convergent evolution (Keim et al., 2009). In a study on the genotyping of 191 *B. anthracis* isolates originating from China, two strains belonging to different canSNP groups (A.Br.Ames and A.Br.001/002) had identical genotypes identified by the MLVA15 scheme (Simonson et al., 2009). Our global *B. anthracis* MLVA database, comprising 559 strains, revealed several examples of homoplasy at VNTR loci in strains belonging to different canSNP groups (see Supplementary Figure S1). In order to minimize the occurrence of misleading results due to homoplasy, Keim et al. (2009) recommended the usage of 13 highly stable and phylogenetically informative canSNPs prior to conducting an MLVA analysis to categorize an unknown isolate into a defined clade. They further proposed the subsequent use of MLVA to effectively discriminate between closely related isolates within a canSNP clade. To avoid the possibility of misidentifying an unknown strain’s closest relatives based solely on MLVA typing outcomes, it is essential to combine our NGS-based MLVA analysis method with the identification of the 13 canSNP markers prior to or parallel with the MLVA analysis. As ONT technology is well-suited to the accurate identification of single nucleotide polymorphisms (SNPs) (Linde et al., 2023), our multiplex PCR-based amplicon sequencing method can be extended to include the identification of canSNPs in a single assay. However, careful primer design is required to ensure the necessary specificity, as environmental samples may be contaminated with *B. cereus* species that are genetically similar to *B. anthracis* in these canSNP regions.

Four of the 11 Hungarian strains analyzed in this study lack one or two of virulence plasmids. The absence of virulence plasmids has been observed in isolates from soil samples (Braun et al., 2015). Additionally, it has been observed that originally virulent *B. anthracis* strains can lose one or both of plasmids during long-term storage in culture collections (Buyuk et al., 2015). It is assumed that the loss of plasmids from the four plasmid-less Hungarian strains occurred during long-term storage conditions, as all the analyzed strains were originally isolated from infected animal hosts. In order to accurately determine the closest relatives and provide an appropriate interpretation of the diversity of Hungarian strains, we did not included markers located on plasmids (pXO1, pXO2, vntr16, vntr17) in the analysis. The pipeline’s module for determining close relatives excludes these markers when analyzing a plasmid-free strain, but includes them when analyzing a strain with a plasmid. Therefore, the calculation of the distance matrix is based on a different number of markers in the two cases, thus resulting in the classification of a plasmid-less and a plasmid-harboring strain with identical chromosomal markers into different groups, as it was observed in strains BaML_9 and BaML_36. As Antwerpen et al. (2011) described, the false clustering resulting from the application of a similar clustering algorithm was observed in an MLVA analysis of Bulgarian *B. anthracis* isolates. Although the MLVA typing system, which uses only chromosomal markers, provides accurate results in epidemiological studies, in preliminary forensic investigations of an unknown *B. anthracis* strain, especially in bioterrorism-related outbreaks, all genotyping data must be involved into analysis (Antwerpen et al., 2011).

The third-generation Oxford Nanopore NGS method is suitable for the sequencing of long, highly repetitive VNTRs by generating long reads which successfully span the repeat regions. However, the method is prone to errors, particularly in homopolymer regions and high GC-containing sequences (Delahaye and Nicolas, 2021), which may affect the accurate determination of repeat numbers by mathematical calculation from the sequence length of VNTR loci. The evolution of the basecallers and the substantial development in flow cells and chemistries has been demonstrated to result in a substantial enhancement of the accuracy of the read sequences and decreased error rates from 7 to 3% (Zhang et al., 2023). To minimize the effects of sequencing errors, we applied the newest V14 chemistry with R10.4.1. flow cells and the latest version of Dorado basecalling software running in “superaccurate basecalling” mode. Moreover, the bioinformatic pipeline incorporates numerous read filtration stages, subsequently followed by the generation of consensus sequences derived from a sufficient number of filtered reads. These sequences are then aligned to the genomes of *B. cereus* group species to ensure specificity. These steps facilitate the exclusion of reads containing errors in repeat regions and being non-specific to *B. anthracis*. Our results show, that the error rates associated with ONT technology did not affect the accuracy of repeat number determination.

The small-size and portable MinION sequencing device has the potential to facilitate the implementation of our *B. anthracis* genotyping method in field laboratories deployed near the infected area, thereby enabling on-site analysis of samples. Field laboratories are used in many organizations, including military defense, intergovernmental and international bodies, public and veterinary health, and law enforcement (Parsons et al., 2018). In the context of biodefense applications, the portable sequencing device MinION was evaluated as part of a rapidly deployable laboratory specialized in the identification of highly pathogenic agents during a NATO live agent exercise in 2016 (Walter et al., 2017). The most significant challenges associated with the application of the MinION sequencer in the field were the preparation of sequenceable libraries from samples with limited DNA yield and complex backgrounds, as well as offline data collection and analysis of raw data due to the lack of the Internet in resource-limited remote deployment sites. The library preparation stage of our recently developed NGS-based *B. anthracis* MLVA genotyping method incorporates a series of additional DNA purification steps, with the objective of generating a high-quality library that yields sufficient sequencing data for subsequent downstream analysis. However, this is still a highly technical and time-consuming process. To adapt this method for field laboratory applications, a shorter and simplified library preparation is required. The availability of new upgrades to the procedure, and using new low-input DNA kits have the potential to simplify and shorten library preparation (Walter et al., 2017).

## 5 Conclusion

The primary advantage of our novel NGS-based MLVA genotyping method is its ability to directly genotype an unidentified *B. anthracis* strain from environmental samples without the need for culturing. Compared to the one-week time requirement of the reference method, the novel method enables a more rapid 24-h turnaround time for genotyping the pathogen from DNA isolated directly from samples collected in contaminated areas. This method can be a powerful tool for field biodefense laboratories to conduct preliminary forensic investigations of bioterrorist-related *B. anthracis* outbreaks, thereby facilitating the establishment of links between cases and identification of the source strain. The data presented herein demonstrate the principles for developing similar ONT amplicon sequencing methods for the rapid genotyping of other bioterrorism-related pathogens.

By determining the MLVA genotypes of 11 Hungarian wild-type *B. anthracis* isolates, the present study provides an initial overview of the genetic diversity of *B. anthracis* in Hungary. The dominant endemic *B. anthracis* population in Hungary comprises strains belonging to the A.Br.008/009 (TEA) clade. Furthermore, our results suggest a prolonged stable presence of the B.Br.CNEVA lineage in Hungary. In future studies, employing whole-genome sequence analysis of more Hungarian *B. anthracis* isolates will further improve the description of the detailed genetic landscape of *B. anthracis* diversity in Hungary and reveal the possible origin of strains.

## Data Availability

The raw sequencing data generated in this study have been deposited in the National Center for Biotechnology Information (NCBI) Sequence Read Archive (SRA) under the Bioproject PRJNA1313341. The datasets presented in this study can be found in online repositories. The names of the repository/repositories and accession number(s) can be found in the article/Supplementary material.

## Footnotes

1 Available online at: https://dx.doi.org/10.17504/protocols.io.3byl4jzozlo5/v3.

2
https://www.ncbi.nlm.nih.gov/tools/primer-blast/

3
https://github.com/nagyagnes/Bant_MLVA31_analyzer

4 Available online at: https://zenodo.org/records/18399429.

5 Available online at: https://zenodo.org/records/18399383.
