# Supplementary material for: Development of Nanopore amplicon sequencing method for culture-free genotyping of Bacillus anthracis strains directly from environmental samples
Source: Front Microbiol. 2026 Mar 13;17:1771578. doi: 10.3389/fmicb.2026.1771578 (PMC13021834; doi:10.3389/fmicb.2026.1771578)
Supplement: Supplementary file 1 [file Data_Sheet_1.PDF]

Table S1: Primer sequences used for *Bacillus anthracis* identification, and sequences of primers designed for identification of 13 canonical SNPs with Delayed Mismatch Amplification Assay (DMAA) method

Bacillus anthracis qPCR primers

Name	Forward	Reverse	Probe	Reference	Amplification scheme
PL3	AAAGCTACAAACTCTGAAATTTGTAAATTG	CAACGATGATTGGAGATAGAGTATTCTTT	FAM-AACAGTACGTTTCACTGGAGCAAAATCAA-IowaBlackFQ	Wielinga et al. 2011	3 min of polymerase activation at 95 °C followed by 45 cycles of 5 s 95 °C, 20 s 62 °C

Wielinga PR, Hamidjaja RA, Agren J, Knutsson R, Segerman B, Fricker M, Ehling-Schulz M, de Groot A, Burton J, Brooks T, Janse I, van Rotterdam B. A multiplex real-time PCR for identifying and differentiating B. anthracis virulent types. Int J Food Microbiol. 2011 Mar 1;145 Suppl 1:S137-44. doi: 10.1016/j.ijfoodmicro.2010.07.039. Epub 2010 Aug 10. PMID: 20826037.

canSNP DMAA primers

canSNP name	SNP locus	Allele base	Derived Forward	Ancestor Forward	Reverse	Amplification scheme
ABr001	182106	C (DER), T (ANC)	AACCAAATTTAATCTTTAAAGGAAAACCGAGAC	AACCAAATTTAATCTTTAAAGGAAAACCGAGAT	TATAATACGGTTTCCCTTTATCATCGAC	3 minutes of polymerase activation at 95 °C, followed by 45 cycles of 5 seconds at 95 °C, 10 seconds at 55 °C, and 20 seconds at 72 °C
ABr002	947760	T (DER), C (ANC)	CAGAAGGAGCAAGTAATGTTATAGGTTTATGT	AGAAGGAGCAAGTAATGTTATAGGTTTATGC	ACCTAAAATCGATAAAGCGACTGC	
ABr003	1493280	C (DER), T (ANC)	GGAATTTAGATTTTCGTGTCGAATTACGC	AGGAATTTAGATTTTCGTGTCGAATTACGT	GCTACTGTCATTGTATAAAAACCTCCT	
ABr004	3600786	C (DER), T (ANC)	CGCCGTCATACTTTGGAACGC	CGCCGTCATACTTTGGAACGT	TGGAATTGGTGGAGCTATGGAA	
ABr006	162509	A (DER), C (ANC)	TCAATATGTTGTTGATCATTCCATCGCTTA	CAATATGTTGTTGATCATTCCATCGCTTC	CAATCTAGCGTTTTTAAGTTCATCATACC	
ABr007	266439	G (DER), A (ANC)	AGGTGGTAGTATTCGAGCTGACTG	CAAGGTGGTAGTATTCGAGCTGACTA	AACGAGACGATAAACTGAATAATACCATC	
ABr008	3947375	C (DER), A (ANC)	GAAAAAGTTACAAATATACGTTTAACAAGCTGC	TGAAAAAGTTACAAATATACGTTTAACAAGCTGA	ACTACGCTATACGTTTTAGATGGAGAT	
ABr009	2589947	G (DER), A (ANC)	CCACTGTTTTTGAACGGCTCTG	GCCACTGTTTTTGAACGGCTCTA	TTTAGGTATATTAACTGCGGATGATGC	
BBr001	1455402	G (DER), A (ANC)	CGGTCATAAAAGAAATCGGTACAATAGAATAG	ACGGTCATAAAAGAAATCGGTACAATAGAATAA	CAAAAGGTTCGGATATGATACCGATAC	
BBr002	1056740	A (DER), C (ANC)	GGAGAAGTTGCAAAAGGAACCGA	GAGAAGTTGCAAAAGGAACCGC	ACCTTCTGTGTTCGTTGTTAACG	
BBr003	1494392	A (DER), G (ANC)	GCATAGAAGCAGATGAGCTTACATATACA	GCATAGAAGCAGATGAGCTTACATATACG	CTCAAGTTCATAACGAACCATAACGT	
BBr004	69952	C (DER), T (ANC)	GCTTGGGTAACCTTCTTTACTTCGAC	GCTTGGGTAACCTTCTTTACTTCGAT	GAAGAATAAAATGAAGATAATGACAAACGG	
ABBr001	3698013	A (DER), G (ANC)	CAATCGCTGCACTCTTTTATTTCGA	CAATCGCTGCACTCTTTTATTTCGG	CGATAATTTTCACAAAGCCGCT	

canSNP DMAA primers were designed by the method described by Antwerpen et al 2019*. Primer sequences are based on the modification of Melt-MAMA primers designed by Birdsell et al. 2012†

*Antwerpen M, Beyer W, Bassy O, Ortega-García MV, Cabria-Ramos JC, Grass G, Wölfel R. Phylogenetic Placement of Isolates Within the Trans-Eurasian Clade A.Br.008/009 of Bacillus anthracis. Microorganisms. 2019; 7(12):689. doi: 10.3390/microorganisms7120689

†Birdsell DN, Pearson T, Price EP, Hornstra HM, Nera RD, et al. (2012) Melt Analysis of Mismatch Amplification Mutation Assays (Melt-MAMA): A Functional Study of a Cost-Effective SNP Genotyping Assay in Bacterial Models. PLoS ONE 7(3): e32866. doi:10.1371/journal.pone.0032866
