# Supplementary material for: Development of Nanopore amplicon sequencing method for culture-free genotyping of Bacillus anthracis strains directly from environmental samples
Source: Front Microbiol. 2026 Mar 13;17:1771578. doi: 10.3389/fmicb.2026.1771578 (PMC13021834; doi:10.3389/fmicb.2026.1771578)
Supplement: Supplementary file 2 [file Data_Sheet_2.PDF]

Table S2: PCR reaction parameters used in capillary electrophoresis (CE)

VNTR marker	PCR assay	Primer concentration (pmol/reaction)	Primer concentration (nM)	Label
vntr23	1	4	200	FAM
vrrA		2	100	FAM
bams25		10	500	FAM
bams24	2	10	500	FAM
bams34		12	600	Yakima Yellow
CG3	3	6	300	FAM
vrrC2		4	200	FAM
bams28		5	250	Yakima Yellow
bams21	4	10	500	Yakima Yellow
bams31		10	500	FAM
vrrB2	5	2	100	FAM
vrrC1		10	500	Yakima Yellow
bams05		6	300	FAM
bams44		6	300	FAM
bams01	6	12	600	FAM
bams53		4	200	FAM
bams22	7	8	400	FAM
bams23		4	200	FAM
bams51		6	300	FAM
vntr19	8	5	250	FAM
vntr12		5	250	FAM
vntr16	9	5	250	FAM
vntr17		5	250	FAM
pXO1	10	5	250	FAM
pXO2	11	5	250	FAM
bams03	12	5	250	FAM
vntr35	13	5	250	FAM
bams30	14	5	250	FAM
bams13	15	5	250	FAM
bams15	16	5	250	Yakima Yellow
vrrB1	17	5	250	FAM

Amplification scheme: 30 sec of polymerase activation at 98 °C followed by 35 cycles of 15 sec 98 °C, 4 min 20 sec 65 °C.
