# Supplementary material for: Development of Nanopore amplicon sequencing method for culture-free genotyping of Bacillus anthracis strains directly from environmental samples
Source: Front Microbiol. 2026 Mar 13;17:1771578. doi: 10.3389/fmicb.2026.1771578 (PMC13021834; doi:10.3389/fmicb.2026.1771578)
Supplement: Supplementary file 3 [file Data_Sheet_3.PDF]

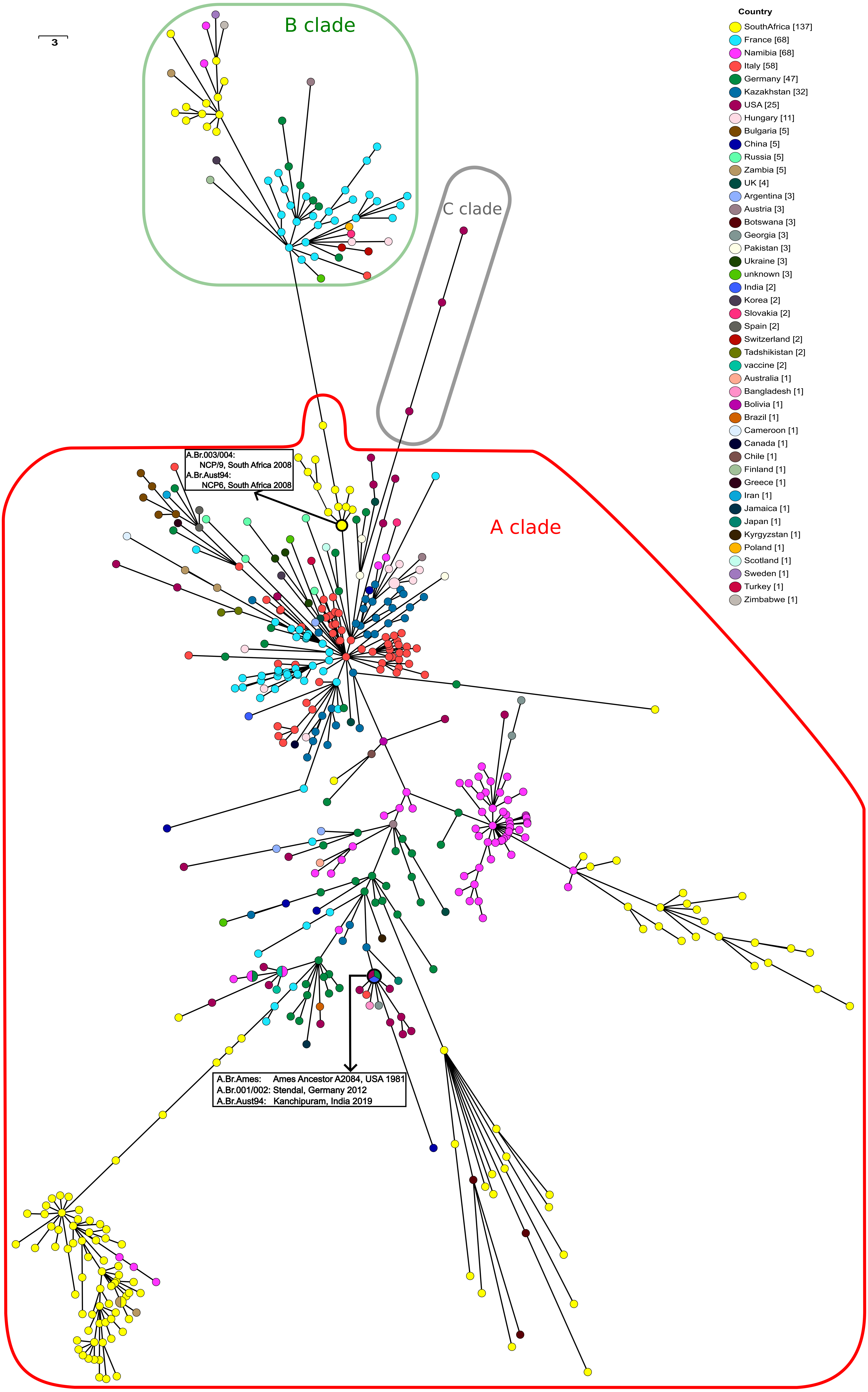

Figure S1: Minimum spanning tree of the MLVA genotypes of 523 Bacillus anthracis strains in the in-house Bacillus anthracis MLVA database. The strains are marked with different colors according to the country of origin (see legend), and the number of representatives from each country is shown in brackets in the legend. The strains shown in the boxes have the same MLVA genotypes but are from different subclades based on the 13 canonical SNP genotypes.
